# Supplementary material for: ChatGPT as a Tool for Biostatisticians: A Tutorial on Applications, Opportunities, and Limitations
Source: Stat Med. 2025 Oct 23;44(23-24):e70263. doi: 10.1002/sim.70263 (PMC12548020; doi:10.1002/sim.70263)
Supplement: Supplementary file 1 — Data S1. Supporting Information. [file SIM-44-0-s001.zip › Supplementary Materials Additional.zip!/3.6_Simulation_Study/3.6_Simulation_Chat10.pdf]

Coverage prob10 - Coverage Probability Simulation

Take the role of an expert biostatistician for this chat. The goal is to perform a simulation study to assess the coverage probability of a confidence interval with nominal coverage 95%. The setting is a case control study design, which is a retrospective observational study that compares individuals with a disease (cases) to those without it (controls) to identify potential risk factors or exposures associated with the disease.

We aim at the coverage probability of an appropriate effect size estimate in a parametric model. Propose a suitable exact confidence interval and investigate its empirical coverage under the null hypothesis of no effect. Use 5000 Monte Carlo runs and the sample sizes must vary between 20 and 40. Provide results for ten different settings with different sample sizes and marginal probabilities. Be consize in your answer and do not provide unnecessary descriptions.

```python
python

import numpy as np
import pandas as pd
import statsmodels.api as sm
from scipy.stats import norm
import itertools
import ace_tools as tools

# Simulation settings
n_simulations = 5000
alpha = 0.05
z_crit = norm.ppf(1 - alpha / 2)

# Sample size settings
sample_sizes = [(cases, controls) for cases, controls in zip(range(20, 30, 1), range(30, 40,
1))][:10]

# Null hypothesis: log(OR) = 0 => OR = 1
true_log_or = 0
true_or = np.exp(true_log_or)

# Simulate function
def simulate_coverage(cases, controls, p_exposure_cases, p_exposure_controls):
    coverages = []
    for _ in range(n_simulations):
        exposure_cases = np.random.binomial(1, p_exposure_cases, cases)
        exposure_controls = np.random.binomial(1, p_exposure_controls, controls)

        y = np.concatenate([np.ones(cases), np.zeros(controls)])
        x = np.concatenate([exposure_cases, exposure_controls])

        try:
            model = sm.Logit(y, sm.add_constant(x)).fit(disp=0)
            log_or = model.params[1]
            se_log_or = model.bse[1]
            ci_lower = log_or - z_crit * se_log_or
            ci_upper = log_or + z_crit * se_log_or
            coverage = (ci_lower <= true_log_or <= ci_upper)
        except:
            coverage = False  # model failed to converge

        coverages.append(coverage)
```

```
    return np.mean(coverages)

# Run simulation for 10 settings
results = []
marginal_probs = np.linspace(0.1, 0.9, 10)  # varying exposure probabilities

for i, (cases, controls) in enumerate(sample_sizes):
    p = marginal_probs[i]
    coverage = simulate_coverage(cases, controls, p, p)
    results.append({
        "Cases": cases,
        "Controls": controls,
        "P(Exposure|Case)": p,
        "P(Exposure|Control)": p,
        "Empirical Coverage": coverage
    })

results_df = pd.DataFrame(results)
tools.display_dataframe_to_user(name="Empirical Coverage Results", dataframe=results_df)
```

STDOUT/STDERR

```
rn("Maximum Likelihood optimization failed to "
/home/sandbox/.local/lib/python3.11/site-packages/statsmodels/base/model.py:604: ConvergenceWarning: Maximum
  warnings.warn("Maximum Likelihood optimization failed to "
/home/sandbox/.local/lib/python3.11/site-packages/statsmodels/base/model.py:604: ConvergenceWarning: Maximum
  warnings.warn("Maximum Likelihood optimization failed to "
/home/sandbox/.local/lib/python3.11/site-packages/statsmodels/base/model.py:604: ConvergenceWarning: Maximum
  warnings.warn("Maximum Likelihood optimization failed to "
/home/sandbox/.local/lib/python3.11/site-packages/statsmodels/base/model.py:604: ConvergenceWarning: Maximum
  warnings.warn("Maximum Likelihood optimization failed to "
/home/sandbox/.local/lib/python3.11/site-packages/statsmodels/base/model.py:604: ConvergenceWarning: Maximum
  warnings.warn("Maximum Likelihood optimization failed to "
/home/sandbox/.local/lib/python3.11/site-packages/statsmodels/base/model.py:604: ConvergenceWarning: Maximum
  warnings.warn("Maximum Likelihood optimization failed to "
/home/sandbox/.local/lib/python3.11/site-packages/statsmodels/base/model.py:604: ConvergenceWarning: Maximum
  warnings.warn("Maximum Likelihood optimization failed to "
/home/sandbox/.local/lib/python3.11/site-packages/statsmodels/base/model.py:604: ConvergenceWarning: Maximum
  warnings.warn("Maximum Likelihood optimization failed to "
/home/sandbox/.local/lib/python3.11/site-packages/statsmodels/base/model.py:604: ConvergenceWarning: Maximum
  warnings.warn("Maximum Likelihood optimization failed to "
/home/sandbox/.local/lib/python3.11/site-packages/statsmodels/base/model.py:604: ConvergenceWarning: Maximum
  warnings.warn("Maximum Likelihood optimization failed to "
/home/sandbox/.local/lib/python3.11/site-packages/statsmodels/base/model.py:604: ConvergenceWarning: Maximum
  warnings.warn("Maximum Likelihood optimization failed to "
/home/sandbox/.local/lib/python3.11/site-packages/statsmodels/base/model.py:604: ConvergenceWarning: Maximum
  warnings.warn("Maximum Likelihood optimization failed to "
/home/sandbox/.local/lib/python3.11/site-packages/statsmodels/base/model.py:604: ConvergenceWarning: Maximum
  warnings.warn("Maximum Likelihood optimization failed to "
/home/sandbox/.local/lib/python3.11/site-packages/statsmodels/base/model.py:604: ConvergenceWarning: Maximum
  warnings.warn("Maximum Likelihood optimization failed to "
/home/sandbox/.local/lib/python3.11/site-packages/statsmodels/base/model.py:604: ConvergenceWarning: Maximum
  warnings.warn("Maximum Likelihood optimization failed to "
/home/sandbox/.local/lib/python3.11/site-packages/statsmodels/base/model.py:604: ConvergenceWarning: Maximum
  warnings.warn("Maximum Likelihood optimization failed to "
/home/sandbox/.local/lib/python3.11/site-packages/statsmodels/base/model.py:604: ConvergenceWarning: Maximum
  warnings.warn("Maximum Likelihood optimization failed to "
/home/sandbox/.local/lib/python3.11/site-packages/statsmodels/base/model.py:604: ConvergenceWarning: Maximum
  warnings.warn("Maximum Likelihood optimization failed to "
/home/sandbox/.local/lib/python3.11/site-packages/statsmodels/base/model.py:604: ConvergenceWarning: Maximum
  warnings.warn("Maximum Likelihood optimization failed to "
/home/sandbox/.local/lib/python3.11/site-packages/statsmodels/base/model.py:604: ConvergenceWarning: Maximum
  warnings.warn("Maximum Likelihood optimization failed to "
/home/sandbox/.local/lib/python3.11/site-packages/statsmodels/base/model.py:604: ConvergenceWarning: Maximum
  warnings.warn("Maximum Likelihood optimization failed to "
/home/sandbox/.local/lib/python3.11/site-packages/statsmodels/base/model.py:604: ConvergenceWarning: Maximum
  warnings.warn("Maximum Likelihood optimization failed to "
/home/sandbox/.local/lib/python3.11/site-packages/statsmodels/base/model.py:604: ConvergenceWarning: Maximum
  warnings.warn("Maximum Likelihood optimization failed to "
/home/sandbox/.local/lib/python3.11/site-packages/statsmodels/base/model.py:604: ConvergenceWarning: Maximum
  warnings.warn("Maximum Likelihood optimization failed to "
/home/sandbox/.local/lib/python3.11/site-packages/statsmodels/base/model.py:604: ConvergenceWarning: Maximum
  warnings.warn("Maximum Likelihood optimization failed to "
/home/sandbox/.local/lib/python3.11/site-packages/statsmodels/base/model.py:604: ConvergenceWarning: Maximum
  warnings.warn("Maximum Likelihood optimization failed to "
```

```
/home/sandbox/.local/lib/python3.11/site-packages/statsmodels/base/model.py:604: ConvergenceWarning: Maximum
  warnings.warn("Maximum Likelihood optimization failed to "
/home/sandbox/.local/lib/python3.11/site-packages/statsmodels/base/model.py:604: ConvergenceWarning: Maximum
  warnings.warn("Maximum Likelihood optimization failed to "
/home/sandbox/.local/lib/python3.11/site-packages/statsmodels/base/model.py:604: ConvergenceWarning: Maximum
  warnings.warn("Maximum Likelihood optimization failed to "
/home/sandbox/.local/lib/python3.11/site-packages/statsmodels/base/model.py:604: ConvergenceWarning: Maximum
  warnings.warn("Maximum Likelihood optimization failed to "
/home/sandbox/.local/lib/python3.11/site-packages/statsmodels/base/model.py:604: ConvergenceWarning: Maximum
  warnings.warn("Maximum Likelihood optimization failed to "
/home/sandbox/.local/lib/python3.11/site-packages/statsmodels/base/model.py:604: ConvergenceWarning: Maximum
  warnings.warn("Maximum Likelihood optimization failed to "
/home/sandbox/.local/lib/python3.11/site-packages/statsmodels/base/model.py:604: ConvergenceWarning: Maximum
  warnings.warn("Maximum Likelihood optimization failed to "
/home/sandbox/.local/lib/python3.11/site-packages/statsmodels/base/model.py:604: ConvergenceWarning: Maximum
  warnings.warn("Maximum Likelihood optimization failed to "
/home/sandbox/.local/lib/python3.11/site-packages/statsmodels/base/model.py:604: ConvergenceWarning: Maximum
  warnings.warn("Maximum Likelihood optimization failed to "
/home/sandbox/.local/lib/python3.11/site-packages/statsmodels/base/model.py:604: ConvergenceWarning: Maximum
  warnings.warn("Maximum Likelihood optimization failed to "
/home/sandbox/.local/lib/python3.11/site-packages/statsmodels/base/model.py:604: ConvergenceWarning: Maximum
  warnings.warn("Maximum Likelihood optimization failed to "
/home/sandbox/.local/lib/python3.11/site-packages/statsmodels/base/model.py:604: ConvergenceWarning: Maximum
  warnings.warn("Maximum Likelihood optimization failed to "
/home/sandbox/.local/lib/python3.11/site-packages/statsmodels/base/model.py:604: ConvergenceWarning: Maximum
  warnings.warn("Maximum Likelihood optimization failed to "
/home/sandbox/.local/lib/python3.11/site-packages/statsmodels/base/model.py:604: ConvergenceWarning: Maximum
  warnings.warn("Maximum Likelihood optimization failed to "
/home/sandbox/.local/lib/python3.11/site-packages/statsmodels/base/model.py:604: ConvergenceWarning: Maximum
  warnings.warn("Maximum Likelihood optimization failed to "
/home/sandbox/.local/lib/python3.11/site-packages/statsmodels/base/model.py:604: ConvergenceWarning: Maximum
  warnings.warn("Maximum Likelihood optimization failed to "
/home/sandbox/.local/lib/python3.11/site-packages/statsmodels/base/model.py:604: ConvergenceWarning: Maximum
  warnings.warn("Maximum Likelihood optimization failed to "
/home/sandbox/.local/lib/python3.11/site-packages/statsmodels/base/model.py:604: ConvergenceWarning: Maximum
  warnings.warn("Maximum Likelihood optimization failed to "
/home/sandbox/.local/lib/python3.11/site-packages/statsmodels/base/model.py:604: ConvergenceWarning: Maximum
  warnings.warn("Maximum Likelihood optimization failed to "
/home/sandbox/.local/lib/python3.11/site-packages/statsmodels/base/model.py:604: ConvergenceWarning: Maximum
  warnings.warn("Maximum Likelihood optimization failed to "
/home/sandbox/.local/lib/python3.11/site-packages/statsmodels/base/model.py:604: ConvergenceWarning: Maximum
  warnings.warn("Maximum Likelihood optimization failed to "
/home/sandbox/.local/lib/python3.11/site-packages/statsmodels/base/model.py:604: ConvergenceWarning: Maximum
  warnings.warn("Maximum Likelihood optimization failed to "
/home/sandbox/.local/lib/python3.11/site-packages/statsmodels/base/model.py:604: ConvergenceWarning: Maximum
  warnings.warn("Maximum Likelihood optimization failed to "
/home/sandbox/.local/lib/python3.11/site-packages/statsmodels/base/model.py:604: ConvergenceWarning: Maximum
  warnings.warn("Maximum Likelihood optimization failed to "
/home/sandbox/.local/lib/python3.11/site-packages/statsmodels/base/model.py:604: ConvergenceWarning: Maximum
  warnings.warn("Maximum Likelihood optimization failed to "
/home/sandbox/.local/lib/python3.11/site-packages/statsmodels/base/model.py:604: ConvergenceWarning: Maximum
  warnings.warn("Maximum Likelihood optimization failed to "
/home/sandbox/.local/lib/python3.11/site-packages/statsmodels/base/model.py:604: ConvergenceWarning: Maximum
  warnings.warn("Maximum Likelihood optimization failed to "
/home/sandbox/.local/lib/python3.11/site-packages/statsmodels/base/model.py:604: ConvergenceWarning: Maximum
  warnings.warn("Maximum Likelihood optimization failed to "
/home/sandbox/.local/lib/python3.11/site-packages/statsmodels/base/model.py:604: ConvergenceWarning: Maximum
  warnings.warn("Maximum Likelihood optimization failed to "
/home/sandbox/.local/lib/python3.11/site-packages/statsmodels/base/model.py:604: ConvergenceWarning: Maximum
  warnings.warn("Maximum Likelihood optimization failed to "
/home/sandbox/.local/lib/python3.11/site-packages/statsmodels/base/model.py:604: ConvergenceWarning: Maximum
  warnings.warn("Maximum Likelihood optimization failed to "
/home/sandbox/.local/lib/python3.11/site-packages/statsmodels/base/model.py:604: ConvergenceWarning: Maximum
  warnings.warn("Maximum Likelihood optimization failed to "
/home/sandbox/.local/lib/python3.11/site-packages/statsmodels/base/model.py:604: ConvergenceWarning: Maximum
  warnings.warn("Maximum Likelihood optimization failed to "
/home/sandbox/.local/lib/python3.11/site-packages/statsmodels/base/model.py:604: ConvergenceWarning: Maximum
  warnings.warn("Maximum Likelihood optimization failed to "
/home/sandbox/.local/lib/python3.11/site-packages/statsmodels/base/model.py:604: ConvergenceWarning: Maximum
  warnings.warn("Maximum Likelihood optimization failed to "
/home/sandbox/.local/lib/python3.11/site-packages/statsmodels/base/model.py:604: ConvergenceWarning: Maximum
  warnings.warn("Maximum Likelihood optimization failed to "
/home/sandbox/.local/lib/python3.11/site-packages/statsmodels/base/model.py:604: ConvergenceWarning: Maximum
  warnings.warn("Maximum Likelihood optimization failed to "
/home/sandbox/.local/lib/python3.11/site-packages/statsmodels/base/model.py:604: ConvergenceWarning: Maximum
  warnings.warn("Maximum Likelihood optimization failed to "
/home/sandbox/.local/lib/python3.11/site-packages/statsmodels/base/model.py:604: ConvergenceWarning: Maximum
  warnings.warn("Maximum Likelihood optimization failed to "
/home/sandbox/.local/lib/python3.11/site-packages/statsmodels/base/model.py:604: ConvergenceWarning: Maximum
  warnings.warn("Maximum Likelihood optimization failed to "
```

```
/home/sandbox/.local/lib/python3.11/site-packages/statsmodels/base/model.py:604: ConvergenceWarning: Maximum
  warnings.warn("Maximum Likelihood optimization failed to "
/home/sandbox/.local/lib/python3.11/site-packages/statsmodels/base/model.py:604: ConvergenceWarning: Maximum
  warnings.warn("Maximum Likelihood optimization failed to "
/home/sandbox/.local/lib/python3.11/site-packages/statsmodels/base/model.py:604: ConvergenceWarning: Maximum
  warnings.warn("Maximum Likelihood optimization failed to "
/home/sandbox/.local/lib/python3.11/site-packages/statsmodels/base/model.py:604: ConvergenceWarning: Maximum
  warnings.warn("Maximum Likelihood optimization failed to "
/home/sandbox/.local/lib/python3.11/site-packages/statsmodels/base/model.py:604: ConvergenceWarning: Maximum
  warnings.warn("Maximum Likelihood optimization failed to "
/home/sandbox/.local/lib/python3.11/site-packages/statsmodels/base/model.py:604: ConvergenceWarning: Maximum
  warnings.warn("Maximum Likelihood optimization failed to "
/home/sandbox/.local/lib/python3.11/site-packages/statsmodels/base/model.py:604: ConvergenceWarning: Maximum
  warnings.warn("Maximum Likelihood optimization failed to "
/home/sandbox/.local/lib/python3.11/site-packages/statsmodels/base/model.py:604: ConvergenceWarning: Maximum
  warnings.warn("Maximum Likelihood optimization failed to "
/home/sandbox/.local/lib/python3.11/site-packages/statsmodels/base/model.py:604: ConvergenceWarning: Maximum
  warnings.warn("Maximum Likelihood optimization failed to "
/home/sandbox/.local/lib/python3.11/site-packages/statsmodels/base/model.py:604: ConvergenceWarning: Maximum
  warnings.warn("Maximum Likelihood optimization failed to "
/home/sandbox/.local/lib/python3.11/site-packages/statsmodels/base/model.py:604: ConvergenceWarning: Maximum
  warnings.warn("Maximum Likelihood optimization failed to "
/home/sandbox/.local/lib/python3.11/site-packages/statsmodels/base/model.py:604: ConvergenceWarning: Maximum
  warnings.warn("Maximum Likelihood optimization failed to "
/home/sandbox/.local/lib/python3.11/site-packages/statsmodels/base/model.py:604: ConvergenceWarning: Maximum
  warnings.warn("Maximum Likelihood optimization failed to "
/home/sandbox/.local/lib/python3.11/site-packages/statsmodels/base/model.py:604: ConvergenceWarning: Maximum
  warnings.warn("Maximum Likelihood optimization failed to "
/home/sandbox/.local/lib/python3.11/site-packages/statsmodels/base/model.py:604: ConvergenceWarning: Maximum
  warnings.warn("Maximum Likelihood optimization failed to "
/home/sandbox/.local/lib/python3.11/site-packages/statsmodels/base/model.py:604: ConvergenceWarning: Maximum
  warnings.warn("Maximum Likelihood optimization failed to "
/home/sandbox/.local/lib/python3.11/site-packages/statsmodels/base/model.py:604: ConvergenceWarning: Maximum
  warnings.warn("Maximum Likelihood optimization failed to "
/home/sandbox/.local/lib/python3.11/site-packages/statsmodels/base/model.py:604: ConvergenceWarning: Maximum
  warnings.warn("Maximum Likelihood optimization failed to "
/home/sandbox/.local/lib/python3.11/site-packages/statsmodels/base/model.py:604: ConvergenceWarning: Maximum
  warnings.warn("Maximum Likelihood optimization failed to "
```

The table shows the empirical coverage probabilities for 95% confidence intervals of the log-odds ratio under the null hypothesis (true OR = 1) across 10 different case-control sample sizes and marginal exposure probabilities. Please review the results in the table to assess how well the interval performs under varying conditions.
